# RNA methyltransferase METTL3 induces intrinsic resistance to gefitinib by combining with MET to regulate PI3K/AKT pathway in lung adenocarcinoma

**DOI:** 10.1111/jcmm.16114

**Published:** 2021-01-24

**Authors:** Fangyan Gao, Qianqian Wang, Chang Zhang, Chen Zhang, Tianyu Qu, Jingya Zhang, Jifu Wei, Renhua Guo

**Affiliations:** ^1^ Department of Oncology The First Affiliated Hospital of Nanjing Medical University Nanjing China; ^2^ Department of Epidemiology and Biostatistics School of Public Health Nanjing Medical University Nanjing China; ^3^ Research Division of Clinical Pharmacology The First Affiliated Hospital of Nanjing Medical University Nanjing China; ^4^ Department of Epidemiology and Biostatistics School of Public Health Southeast University Nanjing China

**Keywords:** gefitinib resistance, lung adenocarcinoma, METTL3, RNA methyltransferase

## Abstract

Clinical research data show that gefitinib greatly improves the progression‐free survival of patients, so it is used in advanced non‐small cell lung cancer patients with EGFR mutation. However, some patients with EGFR sensitive mutations do not have good effects on initial gefitinib treatment, and this mechanism is rarely studied. METTL3, a part of N6‐adenosine‐methyltransferase, has been reported to play an important role in a variety of tumours. In this study, we found that METTL3 is up‐regulated in gefitinib‐resistant tissues compared to gefitinib‐sensitive tissues. Cell function experiments have proved that under the treatment of gefitinib, METTL3 knockdown promotes apoptosis and inhibits proliferation of lung cancer cells. Mechanistic studies have shown that METTL3 combines with MET and causes the PI3K/AKT signalling pathway to be manipulated, which affects the sensitivity of lung cancer cells to gefitinib. Therefore, our research shows that METTL3 can be used as a molecular marker to predict the efficacy of EGFR‐TKI therapy in patients, and METTL3 may be a potential therapeutic target.

## 1 INTRODUCTION

Lung cancer is one of the malignant tumours with the highest lethal rate in the world and the greatest threat to human health and life according to the latest research statistics.^1^ Non‐small cell lung cancer (NSCLC) accounts for 85% of lung cancer, of which about 40% is lung adenocarcinoma (LUAD).^2^ Previous clinical studies have found epidermal growth factor receptor‐tyrosine kinase inhibitor (EGFR‐TKI) represented by gefitinib can greatly improve the progression‐free survival (PFS) of advanced EGFR‐mutant NSCLC, making EGFR‐TKI its standard first choice.^3^, ^4^, ^5^, ^6^ However, there are still about 25% of patients with EGFR sensitive mutations who are not sensitive to initial gefitinib treatment, which deserves further study.^3^, ^7^

In the past few decades, research has found that epigenetics is closely related to tumours.^8^, ^9^, ^10^, ^11^ Epigenetics refers to changes in gene expression levels based on non‐gene sequence changes, such as DNA methylation and chromatin conformation changes, which have been studied in depth. RNA methylation, as a newly discovered post‐transcription epigenetic modification, has received attention in recent years.^12^, ^13^, ^14^ As an important RNA methylation modification, N6‐methyladenosine (m^6^A) is one of the most abundant chemical modifications on eukaryotic mRNA, which can regulate RNA splicing, stability, translation and nucleation.^15^, ^16^, ^17^ There are three major types of enzymes involved in m^6^A methylation of RNA: writers (ie METTL3, METTL14, WTAP, KIAA1429 and METTL16), erasers (ie FTO and ALKBH5) and readers (ie YTHDF1, YTHDF2 and YTHDF3).^18^ m^6^A modification is closely related to the occurrence and development of various tumours, such as breast cancer, glioblastoma and lung cancer.^19^, ^20^, ^21^ As the most important component of the writers complex, METTL3 plays a very important role in the regulation of gene expression.^22^, ^23^ Studies have shown that epigenetics is related to drug resistance, and the relationship between DNA methylation and drug resistance has been widely studied.^24^, ^25^, ^26^ However, there are few reports on RNA methylation and drug resistance.^27^, ^28^ And little is known about the important role of METTL3 in EGFR‐TKI resistance. Therefore, there is an urgent need to study the role of METTL3 in EGFR‐TKI resistance and describe its function and mechanism.

In this study, we demonstrated that the expression of METTL3 is up‐regulated in LUAD tissues resistant to gefitinib. We found that METTL3 influences the expression of MET by integrating with MET to regulate PI3K/AKT pathway, which affect the response of lung cancer cells to gefitinib. Meanwhile, the level of m^6^A decreased in lung cancer cells that METTL3 was knocked down, suggesting that METTL3 may affect the expression of MET through the m^6^A pathway. It suggested that METTL3 could be a potential target to reverse resistance.

## 2 MATERIALS AND METHODS

### 2.1 Tissue samples

A total of 18 LUAD patients were enrolled in the study. Their genetic test reflected that they had either EGFR exon 19 deletion (19DEL) or L858R. The pathological tissues of both groups without medical treatment were divided into sensitivity (n = 9) and resistance (n = 9) by primary efficacy of gefitinib according to follow‐up results. The project was approved by the Research Ethics Committee of Nanjing Medical University (Nanjing, Jiangsu, China), and we obtained written informed consent from all patients.

### 2.2 Cell cultures

Human lung adenocarcinoma cell lines (PC9 cells and H3255 cells) were obtained from the Institute of Biochemistry and Cell Biology at the Chinese Academy of Sciences (Shanghai, China). The cells were cultured in RPMI 1640 medium supplemented with 10% foetal bovine serum (FBS, Gibco, USA) and 1% penicillin‐streptomycin (Gibco, USA) at 37℃ with 5% CO2 in incubators.

### 2.3 Lentivirus transfection

We used short hairpin RNA (shRNA) to knock down the expression of METTL3 in PC9 cells (MOI = 5) and H3255 cells (MOI = 5). The target sequences for sh‐METTL3 and sh‐NC were described in Table S1. PC9 cells and H3255 cells were cultured into 6‐well plates and transfected with 10 µL lentivirus targeting METTL3 or NC. After transfection, cells were selected for 14 days for further studies.

### 2.4 RNA extraction, reverse transcription and qPCR

Total RNA was extracted from tissues or cells with TRIzol reagent (Thermo Fisher Scientific, USA). Then, 1.0 μg of isolated RNA was reverse‐transcribed to cDNA by using PrimeScript™ RT reagent (Takara, Japan) under manufacturer's instructions. Real‐time PCR analysis was performed with SYBR Green (Takara, Japan). According to the manufacturer's instructions, qPCR and data collection were carried out on StepOnePlus RT‐PCR system (Applied Biosystems, USA). β‐ACTIN was used as a housekeeping gene. The primers used in this study were described in Table S1.

### 2.5 Western blot analysis and antibodies

RIPA buffer (Sigma, USA) was used to lyse total cellular proteins. Cells treated with gefitinib were extracted after 6 hours. After ultrasonic cracking, the lysates were quantified using a BCA Protein Assay kit (Pierce, USA). Total protein was separated on 8% SDS‐PAGE gel and transferred to a PVDF membrane (Millipore, USA). A chemiluminescence system (Bio‐Rad, USA) was used to detect signals after incubating antibodies. The primary antibodies we used were anti‐rabbit METTL3 (387 ~ 402 µg/mL, 1:1000 dilution, Abcam, catalog no.ab195352, USA), MET (50 µg/mL, 1:1000 dilution, Cell Signaling Technology, catalog no.8198, USA), EGFR (10 µg/mL, 1:1000 dilution, Cell Signaling Technology, catalog no.4267, USA), p‐EGFR (427 µg/mL, 1:1000 dilution, Cell Signaling Technology, catalog no.3777, USA), AKT (35 µg/mL, 1:1000 dilution, Cell Signaling Technology, catalog no.4691, USA), p‐AKT (91 µg/mL,1:1000 dilution, Cell Signaling Technology, catalog no.4060, USA) and anti‐mouse β‐ACTIN (624 µg/mL, 1:5000 dilution, Cell Signaling Technology, catalog no.3700, USA). The anti‐rabbit (150 µg/mL, 1:3000 dilution) and anti‐mouse (688 µg/mL, 1:3000 dilution) secondary antibodies were purchased from Cell Signaling Technology. β‐ACTIN was used to as an internal control.

### 2.6 CCK8 assay

The survival of cells with gefitinib treatment was assessed by CCK8 assay (Selleck, Shanghai, China). PC9 cells and H3255 cells transfected with sh‐NC or sh‐METTL3 were seeded into 96‐well plates with 3000 cells/well. The next day, the cells were exposed to different concentrations of gefitinib (MedChemExpress, China) for 72 hours. Then, 10 µL of CCK8 was added into each well and incubated at 37°C for 1 hour. The absorbance was measured at 450 nm by an enzyme‐labelled instrument. Set triplicate and repeated three times independently.

### 2.7 Colony‐formation assay

The PC9 cells and H3255 cells transfected with sh‐METTL3 or sh‐NC were placed in 6‐well plates with 500 cells/well. Then, the cells were treated with different concentrations of gefitinib. Change fresh medium with gefitinib every 3 days. Two weeks later, the colonies were fixed in paraformaldehyde, washed with PBS and stained with 0.1% crystal violet (Sigma, St. Louis, MO, USA). The above experiment was performed in triplicate.

### 2.8 Flow cytometric analysis of apoptosis

The PC9 cells and H3255 cells transfected with sh‐METTL3 or sh‐NC were treated with gefitinib and cultured for 72 hours. Then, trypsin without EDTA was used to harvest the cells. The cells were double stained with Annexin V‐Alexa Fluor 647 and propidium iodide using the Annexin V‐Alexa Fluor 647/PI apoptosis detection kit (YEASEN, China). The cells were analysed using a flow cytometer (FACScan, BD Biosciences). Cell apoptosis ratio was analysed by a flow cytometer (FACScan, BD Biosciences). Cells were divided into 4 categories: viable cells, dead cells, early apoptotic cells or apoptotic cells. The relative apoptotic ratios of cells with gefitinib treatment were compared with cells without gefitinib treatment. The experiment was conducted three times independently.

### 2.9 m^6^A dot blot assay

Total RNA was extracted from PC9 cells and H3255 cells transfected with sh‐METTL3 or sh‐NC with TRIzol reagent (Thermo Fisher Scientific, USA). NanoDrop was used to test the concentration of RNA and dilute RNA to 100 ng/µL with RNase‐free water. 657 µL formamide (Sigma, USA), 210 µL 37% formaldehyde solution (Thermo Fisher Scientific, USA) and 133 µL MOPS 10X Solution (Thermo Fisher Scientific, USA) formulated into RNA incubation buffer. Add triple the volume of RNA incubation buffer to RNA solution and incubated at 65°C for 5 mins. Then, add same volume 20X Saline‐Sodium Citrate Solution (Sigma, USA) to solution mentioned above. 50ng, 100 ng, 200 ng and 400 ng RNA buffers were spotted onto a nylon membrane (Bio‐Rad, USA) by Bio‐dot apparatus (Bio‐Rad, USA). RNA was UV crosslinked to the nylon membrane. Then, the membrane was incubated with 0.02% Methylene blue (Sigma, USA) for 1 hour. The membrane was incubated with anti‐m^6^A antibody (1000 µg/mL, 1:1000 dilution, Abcam; catalog no. 208 577) for 12 hours at 4℃.

### 2.10 RNA immunoprecipitation

RNA immunoprecipitation experiments were conducted with Magna RIP RNA‐binding protein immunoprecipitation kit (Millipore, USA). The PC9 cells and H3255 cells were lysed with RIP lysis buffer. Then, the solution was incubated with 5 μg of rabbit polyclonal anti‐METTL3 (387 ~ 402 µg/mL, Abcam, catalog no. ab195352, USA) or non‐immunized rabbit IgG at 4°C overnight. Immunoprecipitated RNA was analysed by quantitative real‐time PCR. The relative levels of MET genes are normalized with IgG level.

### 2.11 Statistical analysis

The data were performed with SPSS software 22.0 (IBM, SPSS, Chicago, IL, USA) and GraphPad Prism 8 (GraphPad Software, La Jolla, CA, USA). Evaluate the correlation between METTL3 and MET using the linear correlation analysis. Student's *t* test was used to compare the difference between groups. *P* value < .05 was considered to indicate the result statistically significant.

## 3 RESULTS

### 3.1 METTL3 is up‐regulated in LUAD tissues resistant to gefitinib

**FIGURE 1:**
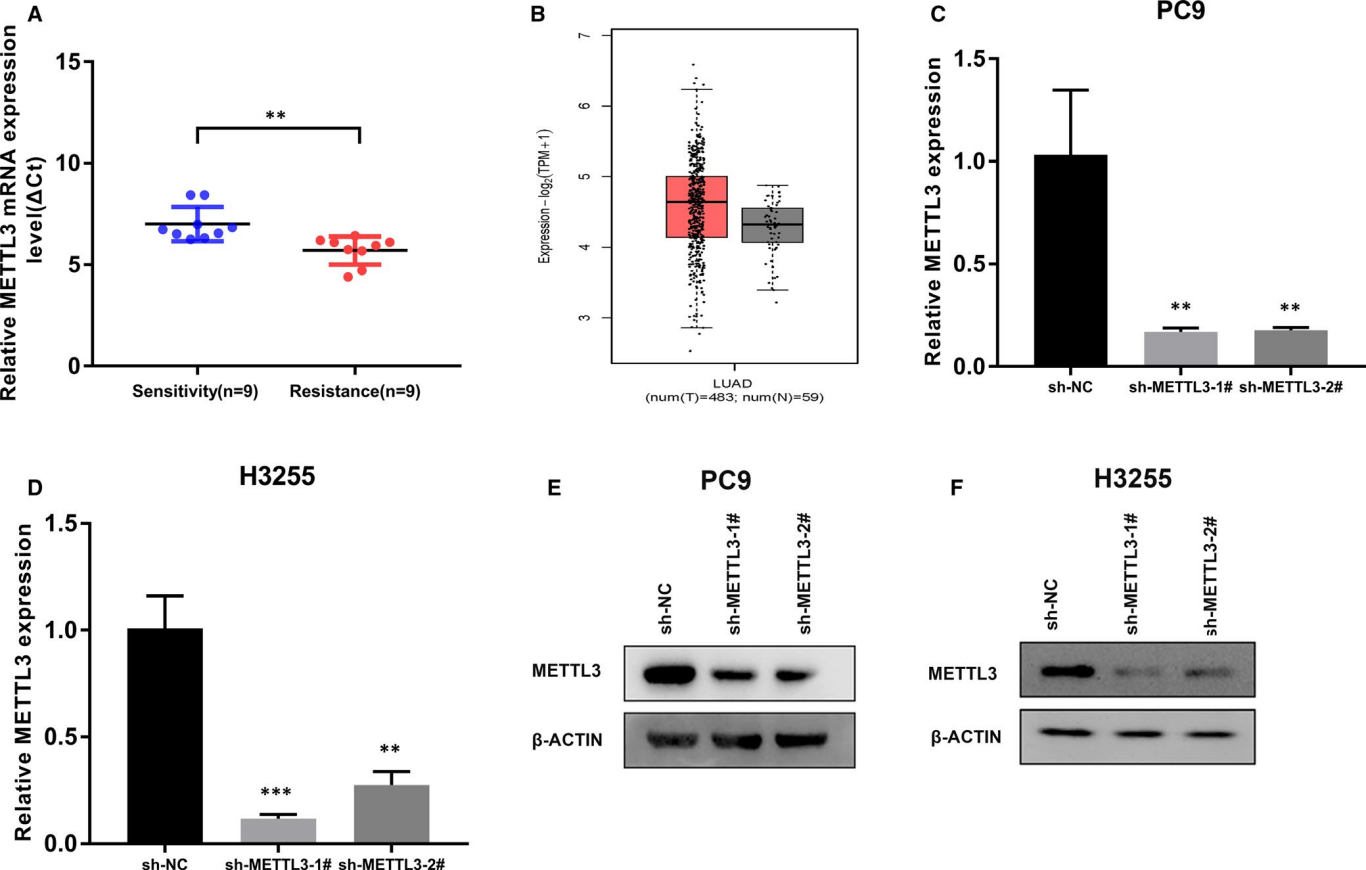
Relative METTL3 expression in LUAD tissues, and the knockdown of METTL3 in cell lines. A, Relative METTL3 mRNA expression in lung adenocarcinoma tissues with EGFR sensitive mutation from unmedicated patients who were sensitive to gefitinib (Sensitivity group) compared with patients who were resistant to gefitinib (Resistance group) after following up results. The expression of METTL3 was measured using qPCR and was normalized to β‐ACTIN expression. The value of ΔCt was calculated by subtracting the β‐ACTIN Ct value from the METTL3 Ct value. Smaller ΔCt values represent higher METTL3 mRNA expression. B, The comparisons of METTL3 mRNA expression in lung adenocarcinoma tissues and normal lung tissues from TCGA database. C and D, Relative METTL3 mRNA expression level in PC9 cells (C) and H3255 cells (D) after transfection with the sh‐NC, sh‐METTL3‐1# or sh‐METTL3‐2#. E and F, METTL3 protein expression level in PC9 cells (E) and H3255 cells (F) after transfection with the sh‐NC, sh‐METTL3‐1# or sh‐METTL3‐2#. The above results are presented as the mean ± SD of three independent experiments. **, *P* < .01, and ***, *P* < .001

In order to study the relationship between METTL3 and gefitinib resistance, we first examined METTL3 mRNA expression in 18 LUAD tissues by quantitative real time (qPCR). The tissues without medical treatment harboured EGFR sensitive mutation: EGFR exon 19 deletion or exon 21 mutation. After following up cases, we divided the tissues into two groups: those who were sensitive to gefitinib (n = 9) and resistant to gefitinib (n = 9). Compared with gefitinib‐sensitive tissues, METTL3 expression was significantly higher (*P < *.01) in gefitinib‐resistant tissues (Figure 1A). Then, we analysed TCGA database and found that METTL3 expression of LUAD tissues exhibited a significant increase compared to normal tissues (Figure 1B). So, the expression of METTL3 is higher in LUAD tissues compared to normal tissues and higher in gefitinib‐resistant LUAD tissues compared to gefitinib‐sensitive LUAD tissues.

### 3.2 Knockdown of METTL3 sensitizes LUAD cells to gefitinib

**FIGURE 2:**
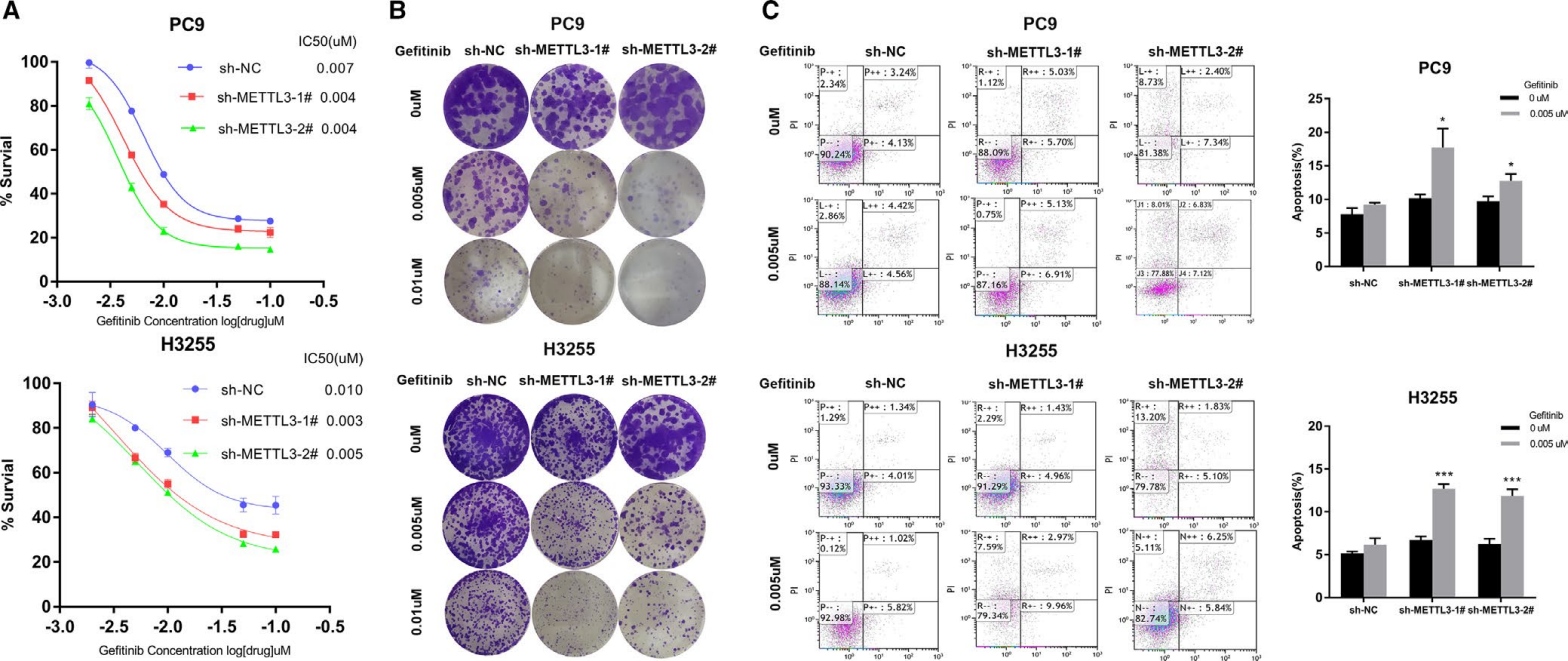
Effect of METTL3 knockdown on the lung adenocarcinoma cell proliferation and cell apoptosis during gefitinib treatment. A, CCK8 assay was used to measure IC50 of transfected PC9 cells and H3255 cells under gefitinib various concentrations treatment for 72 h. B, Assess the cell proliferation by colony‐formation assays of transfected PC9 cells and H3255 cells treated with different concentrations of gefitinib for 14 d. C, Flow cytometric analysis detects the apoptosis of transfected PC9 cells and H3255 cells with or without 0.005 µmol/L gefitinib treatment for 72 h. The above results are presented as the mean ± SD of three independent experiments. *, *P* < .05, and ***, *P* < .001

To study the biological significance of METTL3 in LUAD, PC9 (EGFR exon 19 deletion) cells and H3255 (EGFR exon 21 mutation) cells were chosen for the experiment. Next, we transfected the cells with sh‐NC or two different shRNAs targeting METTL3. qPCR assays show that METTL3 mRNA expression was knocked down to 17.0% and 17.6% in PC9 cells and to 11.7% and 27.4% in H3255 cells (Figure 1C,D). Western blot assays also indicated METTL3 protein significantly decreased in PC9 cells and H3255 cells transfected with shRNA targeting METTL3 (Figure 1E,F). CCK8 assays reflected that PC9 cells and H3255 cells with METTL3 knockdown significantly reduced survival under gefitinib treatment at the same concentration (Figure 2A). Subsequent colony‐formation assays demonstrated that the knockdown of METTL3 reduced colony‐forming ability in the presence of gefitinib compared to control cells (Figure 2B). In addition, flow cytometric analysis reflected that reduced METTL3 expression promotes apoptosis under the treatment with gefitinib (Figure 2C). According to the above result, we found that METTL3 reduce the sensitivity of LUAD to gefitinib.

### 3.3 Knockdown of METTL3 reduced the m^6^A level of total RNAs in PC9 cells and H3255 cells significantly

**FIGURE 3:**
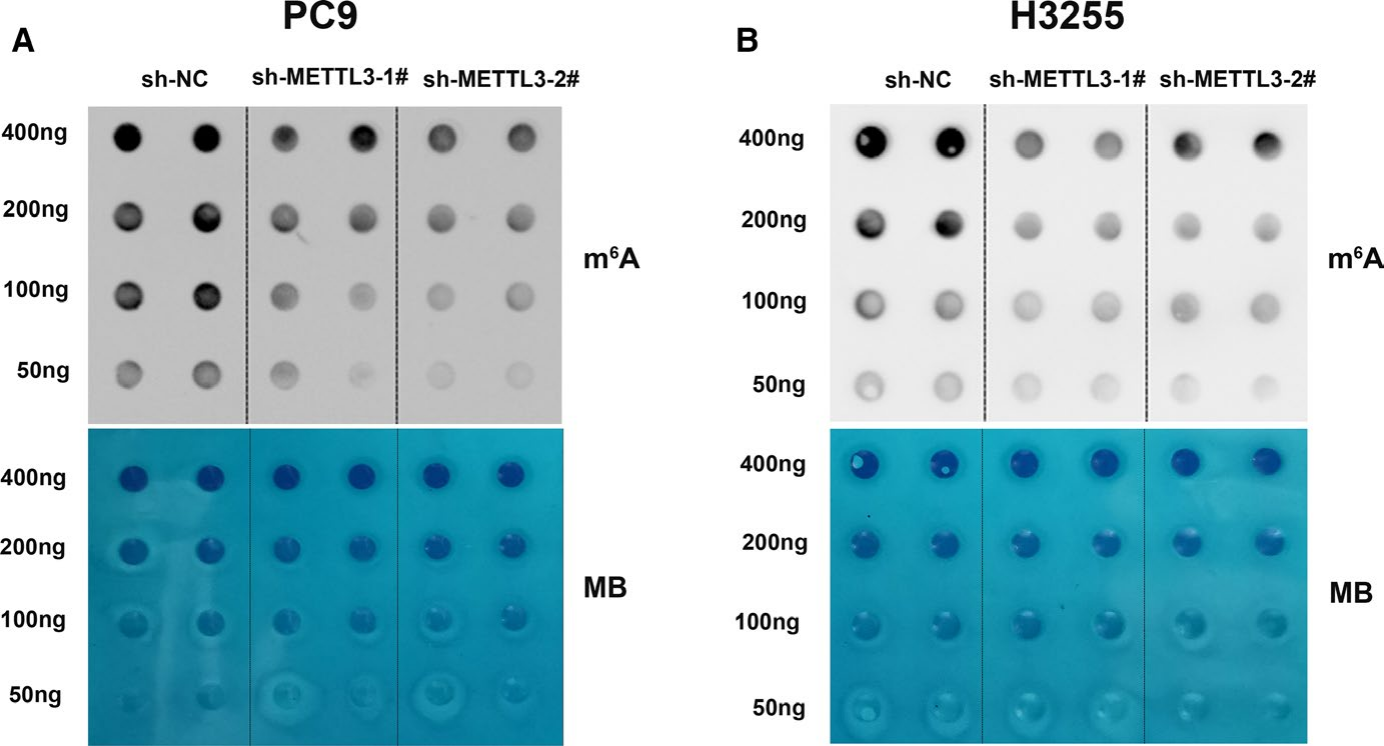
m^6^A Dot Blot Assays of METTL3‐knockdown in PC9 cells and H3255 cells. A and B, m^6^A dot blot assays of PC9 cells and H3255 cells transfected with sh‐NC, the sh‐METTL3‐1# or sh‐METTL3‐2#. MB, methylene blue staining for loading control

To test the change of m^6^A level of total RNAs, we conducted m^6^A dot blot assays. The result reflected that the m^6^A level of total RNAs reduced significantly in PC9 cells and H3255 cells transfected with sh‐METTL3 compared to control groups (Figure 3). Therefore, we speculated that the sensitivity of PC9 cells and H3255 cells transfected with sh‐METTL3 to gefitinib may be related to the changes in m^6^A levels of total RNA.

### 3.4 METTL3 regulated the expression of MET positively and activate the PI3K/AKT pathway to reduce sensitivity to gefitinib

**FIGURE 4:**
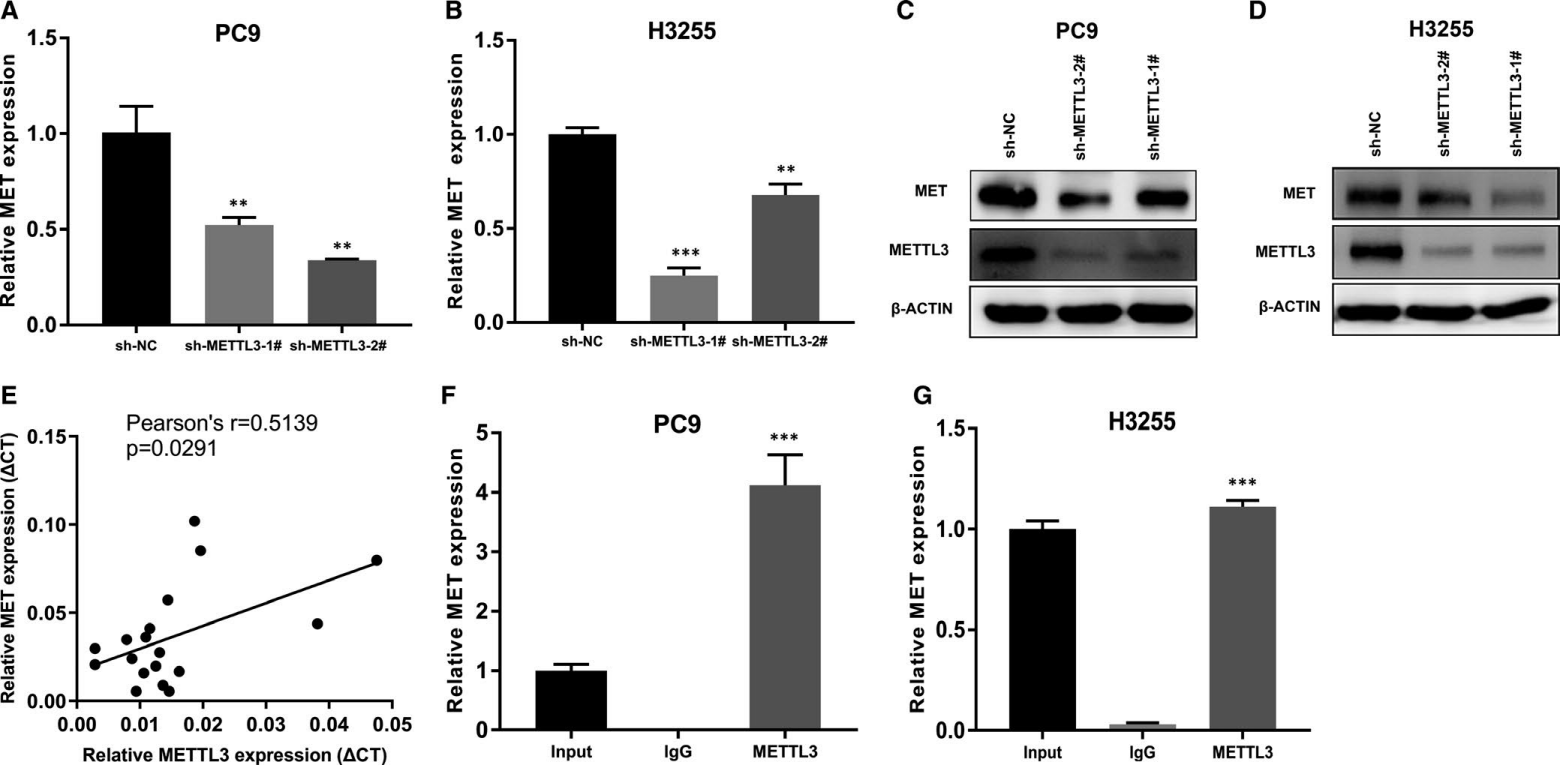
METTL3 is involved in MET up‐regulation. A and B, qPCR analysis of MET expression in PC9 cells and H3255 cells transfected with sh‐NC, sh‐METTL3‐1# or sh‐METTL3‐2#. C and D, Western blot assay was used to detect the level of MET in PC9 cells and H3255 cells after shRNA treatment targeting METTL3 or NC. E, qPCR assay was used to analyse the relationship between MET expression (rCt value) and METTL3 expression (rCt value) in the 18 LUAD tissues mentioned above. F and G, qPCR analysis of immunoprecipitation with METTL3 antibody in PC9 cells and H3255 cells to assess the expression of MET. The above results are mean ± SD of three independent experiments performed in triplicate. **, *P* < .01, and ***, *P* < .001

**FIGURE 5:**
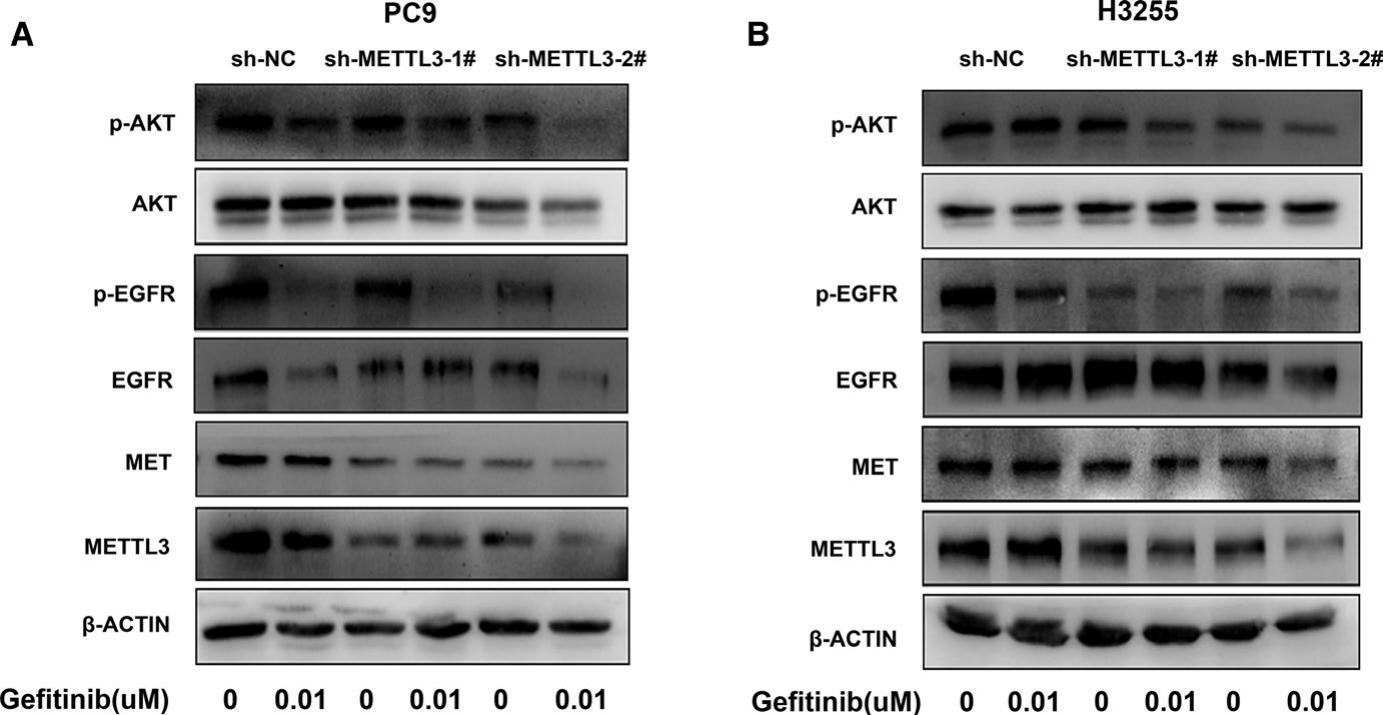
METTL3 regulates PI3K/AKT pathway through MET. A and B, Western blot assays confirmed PI3K/AKT pathway change after transfection with sh‐METTL3 or sh‐NC in PC9 cells and H3255 cells. β‐ACTIN protein was used as an internal control. Representative results were from three independent experiments

To investigate the mechanism of METTL3 in decreasing sensitivity to gefitinib in PC9 cells and H3255 cells, we checked published methylated RNA immunoprecipitation sequencing and data analysis in lung cancer. We found that MET had m^6^A peaks identified in H1299 cell.^22^ According to previous reports, MET is related to primary gefitinib resistance.^29^ So, we conjectured that METTL3 regulated MET expression. qPCR demonstrated our conjecture that the expression of MET decreased significantly after METTL3 knockdown (Figure 4A,B). Western blot assays also show that the knockdown of METTL3 caused a significant decrease in MET protein expression (Figure 4C,D). Next, we verified in the patient tissues. We tested the expression of METTL3 and MET in the 18 tissues mentioned above by qPCR. The result showed that the expression of MET is positively correlated with the expression of METTL3 (Pearson's *r* = .5139, *P* = .0291) in 18 tissues (Figure 4E). In addition, immunoprecipitation assays confirmed that METTL3 interact with MET in PC9 cells and H3255 cells (Figure 4F,G). Treatment of drug‐resistant cells with gefitinib, MET expansion still led to activation of PI3K/AKT signalling pathway and caused cell resistance when EGFR phosphorylation is suppressed.^29^, ^30^ To explore the role of METTL3 in primary gefitinib resistance, we examined the PI3K/AKT pathway which is related with lung cancer growth and resistance. Western blot assays showed that gefitinib can inhibit the phosphorylation of EGFR with or without knocking down METTL3. However, treatment of METTL3‐knockdown cells with gefitinib significantly reduced AKT activation compared to PC9 cells and H3255 cells (Figure 5A,B). The result suggested METTL3 knockdown can reduce AKT activation by decreasing MET, affecting the response to gefitinib.

## 4 DISCUSSION

Gefitinib is a small molecule that targets EGFR and has been successfully used as a first‐line treatment for NSCLC patients with EGFR mutations. The use of targeted therapy has greatly improved the survival rate of NSCLC patients. However, some patients with EGFR mutations are still insensitive to gefitinib.^3^ The molecular mechanism of initial gefitinib resistance in NSCLC patients remains unclear.

In recent years, m^6^A modification is closely related to the occurrence and development of lung cancer.^31^, ^32^, ^33^ Growing evidence has revealed that m^6^A modification is associated with drug resistance.^34^, ^35^, ^36^ It was reported that METTL3 knockdown enhances sensitivity to cisplatin.^37^ However, the relationship between m^6^A modification and EGFR‐TKI resistance is not studied. In this study, we found that the relative expression level of METTL3 is higher in gefitinib‐resistant tissues compared to gefitinib‐sensitive tissues. Knockdown of METTL3 sensitized PC9 cells and H3255 cells to gefitinib, leading to cell apoptosis. Whether the expression of METTL3 is related to the response to gefitinib and prognosis need further study. Meanwhile, we found that knockdown of METTL3 was accompanied by a decreased expression of MET. In patient tissues, we also found METTL3 and MET are positively related. Furthermore, RNA immunoprecipitation (RIP) assays showed that METTL3 could bind MET in PC9 cells and H3255 cells. m^6^A dot blot assays reflected the level of m^6^A in cells with METTL3 knockdown decreased significantly. METTL3, an important RNA methyltransferase, we conjectured that METTL3 may regulate MET through the m^6^A modification. MET was reported to be associated with resistance to gefitinib by activating PI3K/AKT signalling pathway.^7^ In sensitive cells, gefitinib can inhibit EGFR phosphorylation, leading to inactivation of PI3K/AKT signalling. However, in resistant cells, even if gefitinib inhibits the phosphorylation of EGFR, the PI3K/AKT signalling pathway is still activated by MET amplification.^29^ In this study, we found that knockdown of METTL3 also decreased the expression of MET and phosphorylation‐AKT compared to control cells with gefitinib treatment. It suggested that METTL3 could affect the PI3K/AKT signalling pathway by regulating the expression of MET and leads to TKI resistance.

In conclusion, our experiment indicated that METTL3 may be a potential molecular marker to predict patients' efficacy on EGFR‐TKI drugs and METTL3 may be a target to overcome the intrinsic resistance to EGFR‐TKI.

## CONFLICT OF INTEREST

The authors declare no competing interests.

## AUTHOR CONTRIBUTIONS

**Fangyan Gao:** Conceptualization (lead); Data curation (lead); Writing‐original draft (lead). **Qianqian Wang:** Conceptualization (equal); Data curation (equal); Writing‐original draft (equal). **chang zhang:** Data curation (equal); Project administration (equal). **Chen Zhang:** Data curation (equal); Software (equal). **Tianyu Qu:** Project administration (supporting). **Jingya Zhang:** Project administration (equal). **Ji‐Fu Wei:** Supervision (equal); Writing‐review & editing (equal). **Renhua Guo:** Conceptualization (equal); Supervision (lead); Writing‐review & editing (equal).

## Supporting information

Table S1

## Data Availability

The data are free access to available upon request.
